# Novel bacterial taxa in a minimal lignocellulolytic consortium and their potential for lignin and plastics transformation

**DOI:** 10.1038/s43705-022-00176-7

**Published:** 2022-09-26

**Authors:** Carlos Andrés Díaz Rodríguez, Laura Díaz-García, Boyke Bunk, Cathrin Spröer, Katherine Herrera, Natalia A. Tarazona, Luis M. Rodriguez-R, Jörg Overmann, Diego Javier Jiménez

**Affiliations:** 1 Microbiomes and Bioenergy Research Group, Department of Biological Sciences, Universidad de los Andes, Bogotá, Colombia; 2 Department of Chemical and Biological Engineering, Advanced Biomanufacturing Centre, University of Sheffield, Sheffield, UK; 3 Leibniz Institute DSMZ-German Collection of Microorganisms and Cell Cultures, Braunschweig, Germany; 4 Department of Civil and Environmental Engineering, Universidad de los Andes, Bogotá, Colombia; 5 Institute of Active Polymers, Helmholtz-Zentrum Hereon, Teltow, Germany; 6 Department of Microbiology and Digital Science Center (DiSC), University of Innsbruck, Innsbruck, Austria; 7 Braunschweig University of Technology, Braunschweig, Germany

**Keywords:** Metagenomics, Applied microbiology, Microbial ecology, Metagenomics, Soil microbiology

## Abstract

The understanding and manipulation of microbial communities toward the conversion of lignocellulose and plastics are topics of interest in microbial ecology and biotechnology. In this study, the polymer-degrading capability of a minimal lignocellulolytic microbial consortium (MELMC) was explored by genome-resolved metagenomics. The MELMC was mostly composed (>90%) of three bacterial members (*Pseudomonas protegens*; *Pristimantibacillus lignocellulolyticus* gen. nov., sp. nov; and *Ochrobactrum gambitense* sp. nov) recognized by their high-quality metagenome-assembled genomes (MAGs). Functional annotation of these MAGs revealed that *Pr. lignocellulolyticus* could be involved in cellulose and xylan deconstruction, whereas *Ps. protegens* could catabolize lignin-derived chemical compounds. The capacity of the MELMC to transform synthetic plastics was assessed by two strategies: (i) annotation of MAGs against databases containing plastic-transforming enzymes; and (ii) predicting enzymatic activity based on chemical structural similarities between lignin- and plastics-derived chemical compounds, using Simplified Molecular-Input Line-Entry System and Tanimoto coefficients. Enzymes involved in the depolymerization of polyurethane and polybutylene adipate terephthalate were found to be encoded by *Ps. protegens*, which could catabolize phthalates and terephthalic acid. The axenic culture of *Ps. protegens* grew on polyhydroxyalkanoate (PHA) nanoparticles and might be a suitable species for the industrial production of PHAs in the context of lignin and plastic upcycling.

## Introduction

Currently, the energy transition from fossil to renewable resources and the degradation and recycling of plastics are topics of global concern [1, 2]. Lignocellulose is one of the main renewable resources used for the production of biofuels, bioplastics, and other commodity chemicals [3, 4]. In biorefineries, one of the major bottlenecks is the low efficiency in the release of sugar monomers from agricultural residues [5]. Therefore, the use of enzyme cocktails, produced by mixed microbial cultures, has been proposed as an alternative to improve this saccharification process [6]. In this context, the design and characterization of lignocellulolytic microbial consortia has received increasing attention during the last decade [7–10]. These microbial communities can be assembled by two main approaches [11]: (1) the “top-down” enrichment, which consists of the selection of nature-derived populations with the capacity to grow in a minimal medium containing plant biomass as the sole carbon source [12, 13]; and (2) the “bottom-up” strategy, where microbial axenic strains are mixed in different proportions and types to produce a synthetic community [14, 15]. The outcomes of these two approaches are lignocellulolytic microbial consortia that can be characterized by different meta-omics approaches [8, 16–18], In some cases, metagenome-assembled genomes (MAGs) could be retrieved and analyzed from these consortia [19, 20]. In the “top-down” approach, many factors can shape the final selected microbial consortia, including the starting inoculum [21] and the carbon source [22]. In many cases, the microbial communities selected by this enrichment approach are still highly complex, with hundreds of species coexisting in the same flask [16]. These species can interact by synergism, competition, and commensalism, having preferences for certain niches that allow them to coexist in a highly dynamic environment [23]. Inspired by Kang et al. [24], a method to select the minimal number of bacterial species that are able to grow on lignocellulose was recently developed by our research group [13].

Lignocellulolytic microbial communities harbor a huge potential to deconstruct cellulose, xylan, and lignin [25]. Recently, specific lignin-degrading microbial consortia have been developed [26, 27]. Lignin is a heterogeneous aromatic plant polymer formed via radical coupling reactions involving p-coumaryl, coniferyl, and sinapyl alcohols, linked by C–C and C–O bonds [28]. Interestingly, these are the same type of bonds found in the backbone of some fossil- (e.g., polyethylene terephthalate (PET)) and bio-based plastics (e.g., polyhydroxyalkanoates (PHAs)). Therefore, it has been suggested that microbes thriving on lignin (or lignocellulose) could have an enormous enzymatic potential to depolymerize/catabolize plastics and their derived chemical compounds [29, 30]. Particularly, *Pseudomonas*, *Bacillus*, and *Paenibacillus* species, which are found in different lignocellulose-degrading microbial communities [13, 21, 22], could have the capacity to metabolize PET and/or polypropylene [31, 32]. Despite the structural similarities of some plastics and plant-derived polymers, the potential for the transformation of fossil and bio-based plastics by lignocellulose-degrading microbial consortia remains underexplored. The main goal of the current study was to elucidate the polymer-transforming capacity of a minimal and effective lignocellulolytic microbial consortium (hereafter MELMC) [13]. We hypothesized that the MELMC members with the capacity to catabolize lignin, and its derived aromatic compounds, might as well have a big potential to transform fossil- and bio-based plastics. In this study, PacBio-HiFi metagenome sequencing allowed the complete reconstruction of circular and high-quality genomes (i.e., MAGs) of the three most abundant MELMC members. Notably, two of them were analyzed and cataloged as novel bacterial taxa. Through combined use genomic databases and wet-lab experiments, we predicted the functional role of each MELMC member and their capacity to degrade plant polymers, catabolize lignin and transform plastics, as well as their derived chemical compounds.

## Materials and methods

### Construction of a minimal and effective lignocellulolytic bacterial consortium

In 2021, Díaz-García et al. [13] designed and performed an innovative “top-down” strategy to select a MELMC from Colombian Andean Forest soils (Gámbita, Santander). Briefly, the “dilution-to-stimulation” approach was set up to promote the growth of lignocellulose-degrading microorganisms through cultivation under aerobic and mesophilic conditions using a mixture of three agricultural residues (sugarcane bagasse, corn stover, and rice husk) as the sole carbon source. Afterward, the authors executed the “dilution-to-extinction” phase to reduce the microbial diversity through an array of serial dilutions, aiming to select the MELMC. Bacterial 16S rRNA amplicon sequencing was performed to characterize the MELMC, which was the starting point of our current study.

### Isolation and characterization of axenic cultures from the MELMC

The isolation of bacterial pure cultures from the MELMC was done on R2A, Cetrimide, and LB agar (Merck, Darmstadt, Germany). Serial dilutions of the MELMC were done in sterile 0.85% NaCl solution, and 100 μl of each dilution (10^−6^–10^−8^) were spread on the surface of solid media. Morphologically different colonies were selected, purified, and preserved at −80 °C in LB broth with glycerol (20% v/v). Genomic DNA from pure cultures was extracted using the DNeasy UltraClean® Microbial Kit (Qiagen, Hilden, Germany) according to the instruction of the manufacturer. Bacterial 16S rRNA genes were amplified using primers 27F and 1492R. PCR reactions were done in a 50 μl reaction mixture containing 1X OneTaq® DNA Polymerase Quick-Load Master Mix (NEB, Massachusetts, United States) with standard buffer, 0.2 μM of each primer and 100–500 ng of bacterial DNA. The PCR settings are described by Jiménez et al. [12]. PCR products were purified and then sequenced by Sanger Technology using the primer 27F in Macrogen Company (Seoul, Korea). High-quality sequences for each axenic culture were taxonomically affiliated using BLASTn against the NCBI GenBank database (accessed in November 2021).

### Whole-metagenome sequencing and reconstruction of bacterial genomes

Total genomic DNA extracted from the MELMC was used for whole-metagenome sequencing using PacBio Technology. A SMRTbell® template library was prepared according to the instructions from Pacific Biosciences (Menlo Park, CA, United States), following the procedure and checklist—preparing 10 kb library using SMRTbell® Express Template Prep Kit 2.0 for metagenomics shotgun sequencing. Libraries were sequenced on the Sequel*IIe* Instrument, at the Leibniz Institute DSMZ, taking one 30 h movie per SMRT cell. For each sample, 1.33 SMRT cells were run. The raw sequence reads obtained were assembled using the software Flye v2.8 [33] with the metagenome and --pacbio-hifi options. Circular contigs with a length greater than 1.5 Mbp were retained for further analysis. CheckM v1.0.13 [34] was used with the lineage_wf option to assess the quality, completeness, and contamination of the selected contigs. The number of mapped reads from each MAG was calculated using Bowtie v2.3.5 [35]. Curation and structural annotation of MAGs based on rRNAs genes (e.g., 5S, 16S, and 23S) and the *rpoB* gene was carried out with the DFAST v1.2.6 pipeline [36]. The quality assessment percentages of MAGs (i.e., completeness and contamination) were compared to those established by Bowers et al. [37]. The webserver CGView [38] was used to set up a circular map analysis of each MAG, including identification of their genomic islands and phage sequences, which were retrieved using Island Viewer 4 [39] and PHASTER [40] (accessed in January 2022), respectively.

### Taxonomic/phylogenomic placement of the metagenome-assembled genomes

The webserver JSpecies was used to determine a genome distance metric based on the average nucleotide identity (ANI) against related bacterial species [41]. In addition, average amino acid identity (AAI) against closely related genomes was calculated using the Microbial Genomes Atlas (MiGA) v1.2.6.0 [42] against the TypeMat database release r2022-04 [43]. The assessment of novel taxa was considered the ANI and AAI thresholds proposed by Konstantinidis et al. [44] (e.g., new species less than 95% ANI with its closest relatives and a new genus less than 65% AAI with related genera). In addition, the high-quality MAGs were uploaded to the Type (Strain) Genome Server (TYGS) in order to get additional genome features, such as digital DNA–DNA Hybridization (dDDH) and other ANI values [45]. Using the TGYS, phylogenetic comparisons (based on bacterial 16S rRNA gene sequences) and genome/proteome-based phylogenomic analysis (using Genome BLAST Distance Phylogeny method (GBDP)) were carried out [46, 47]. Additional genomic features, including length, G+C content, G-C and A-T skews, protein counts, coding density, and 16S rRNA genes, were obtained by both MiGA and TYGS. The similarity among 16S rRNA gene sequences obtained from the bacterial isolates (~800 bp), retrieved MAGs (~1500 bp), and amplicon-sequence variants (ASVs) (~300 bp), previously obtained by Díaz-García et al. [13] was assessed by a multiple alignment using ClustalW and phylogenetic analysis (Neighbor-Joining, using p-distance method) conducted using the software MEGAX v10.2.6 [48].

### Functional annotation of the metagenome-assembled genomes

Different strategies were used to annotate enzymes associated with carbohydrates, lignin, lipids, and plastic transformation within the MAGs. Annotation of carbohydrate-active enzymes (CAZymes) was performed using the standalone version of dbCAN2 tool v10 [49]. Results were displayed through a heatmap elaborated using the superheat package in R studio software [50]. Data were normalized by the total number of genes associated with each CAZyme family within each MAG, and compared to different reported bacterial genomes. Then, lignin-transforming enzymes were selected based on Diaz-Garcia et al. [51], and the respective genes (i.e., KO identifiers) were searched by the annotation carried out using the RAST [52] and BlastKOALA servers [53]. Finally, the MAGs were aligned using DIAMOND v0.9 [54] against the Plastics Microbial Biodegradation Database (PMBD) [55], and annotated with the PlasticDB [56], the PHA Depolymerase Engineering [57], and the Lipase Engineering [58] databases, setting a minimum amino acid identity cutoff of 50%.

### Construction of a tripartite graph for predicting plastic-transforming enzymes

Information about lignin-transforming enzymes and lignin-derived chemical compounds (LDCC) were retrieved from the eLignin [59] and KEGG databases. Moreover, plastic-derived chemical compounds (PDCC) were selected using the PMBD database [55]. The Simplified Molecular-Input Line-Entry System (SMILES) format of each chemical compound (i.e., LDCC and PDCC) was retrieved using the PubChem database [60]. Based on LDCC and PDCC SMILES, the molecular fingerprints and Tanimoto coefficient [61] for pairs of chemical compounds were calculated using the python RDkit library [62], resulting in a similarity matrix. Chemical compounds with a similarity value greater than or equal to 65% were selected to build an unweighted undirected tripartite graph, using the R Igraph library [63], in which nodes of each partition represent the PDCC connected to the most similar LDCC, and the enzymes that could be involved into the catalysis of the latter, which could by association have an activity of the PDCC.

### Growth of *Ps*. *protegens* using polyhydroxyalkanoates nanoparticles

Given that chemical structure in PHAs can differ and affect the enzymatic catalysis, the ability of *Ps. protegens* (MAG1) to grow on nanoparticles of polyhydroxybutyrate (PHB) (obtained from Sigma Aldrich – CAS 29435-48-1) and polyhydroxyoctanoate (PHO – 98% purity) as the sole carbon source was tested. Briefly, 100 μl of an overnight culture of the *Ps. protegens* (in LB liquid medium) were inoculated in a 100-ml Erlenmeyer flask that contained 20 ml of Minimal-Medium M9 (MM), composed of (g/l) 3 KH_2_PO_2_, 7 (NH_4_)_2_SO_4_, 0.5 NaCl, 1 (NH_4_)Cl and supplemented with 1 mM MgSO_4_, 0.1 mM CaCl_2_ and 20 µl of 1000X trace element solution [64]. In addition, the liquid MM medium contained a final concentration of 0.05 mg/ml PHB or 0.08 mg/ml PHO nanoparticles (emulsion in distilled water) that were added after 20 min UV (254 nm wavelength) sterilization. For the preparation of nanoparticles (100–500 µM), we follow the methodology reported by Schirmer et al. [65]. The controls used for this experiment were: MM + PHB, MM + PHO, both without bacterial inoculum and MM + bacterial inoculum (initial optical density (OD) at 600 nm of 0.03), without PHAs nanoparticles. Duplicate cultures were incubated at 30 °C and 200 rpm. The OD_600nm_ was measured at 48 and 144 h after incubation. Once the cultures reached OD_600nm_ > 1, 100 µl of the bacterial culture were transferred to a fresh liquid MM + PHB/O nanoparticles (pass 2), and then incubated in the above conditions.

## Results

### Features of the metagenome-assembled genomes obtained from the MELMC

**Fig. 1:**
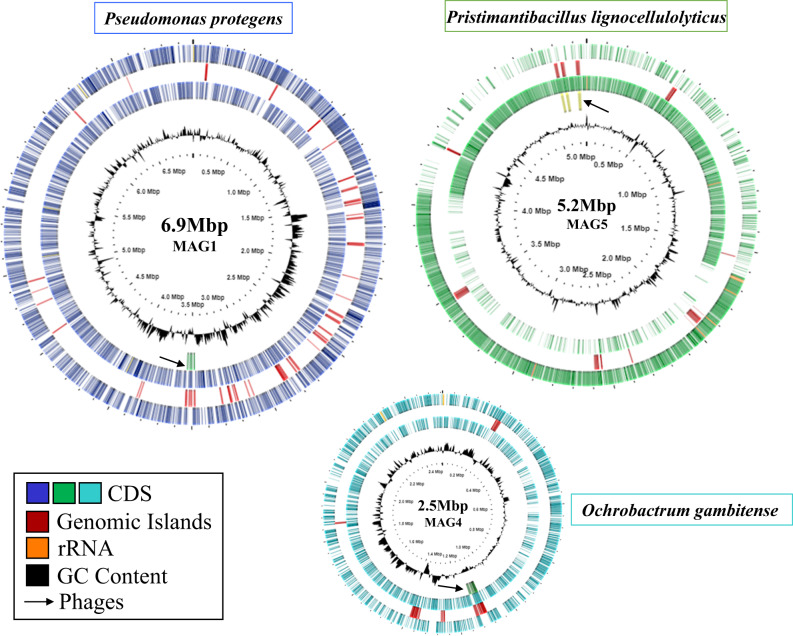
Circular visualization of the MAGs recovered from the MELMC. Circles from outside inwards represent: Circle 1. Protein-coding genes (CDS) in purple for MAG1 (*Pseudomonas protegens*), green for MAG5 (*Pristimantibacillus lignocellulolyticus*), and blue for MAG4 (*Ochrobactrum gambitense*). Circle 2. Genomic islands in red. Circle 3. Ribosomal RNA genes in orange. Circle 4. Phage sequences in green and pointed out with an arrow. Circle 5. GC content in black.

For PacBio-HiFi metagenome sequencing, three biological replicates from the MELMC were selected. For each replicate, around 2.7 million reads were obtained with sizes of 6–7 kb. After merging and assembling all sequence reads, three complete circular and high-quality genomes with sizes of 6.98, 5.26, and 2.52 Mbp were obtained (Fig. 1). The three MAGs recovered showed values for completeness and contamination of 99% and 1.4% (MAG1); 98% and 0.7% (MAG5); 90 and 0% (MAG4). Other MAG features (e.g., mol% GC, coding density, number of 16S rRNA copies, and proteins) can be found in Table 1. Interestingly, some MAGs regions were associated with prophage sequences (Fig. 1 and Supplementary Table 1).

**Table 1:** Features of the reconstructed metagenome-assembled genomes (MAGs) from the MELMC, and their possible taxonomic origin.

	MAG1	MAG5	MAG4
Total length (Mbp)	6.98	5.27	2.53
G+C content (TYGS and MiGA)	63.4%	37.8%	57.2%
Average G-C skew (MiGA)	0.300	0.113	–0.408
Average A-T skew (MiGA)	–0.431	–0.033	–0.015
Number of 16S rRNA copies	5	8	2
Protein count (TYGS and MiGA)	6.197	4.576	2.372
Coding density (TYGS and MiGA)	88.6%	85.5%	86.6%
Number of CAZymes (dbCAN)	204	234	97
Best BlastN hit for 16S rRNA (% identity/QC/vs Taxa-AN)	99.7/99/*Pseudomonas protegens*-NR_114749.1	95.1/99/*Paenibacillus castanea*e-NR_044403.1	98.4/99/*Brucella anthropi-*NR_074243.1
ANIb (%/vs Taxa)	98.44/*Pseudomonas protegens* CHA0	68.05/*Paenibacillus crassostreae* LPB0068	90.32/*Ochrobactrum soli* BO-7
ANIm (%/vs Taxa)	98.91/*Pseudomonas protegens* CHA0	87.70/*Paenibacillus popilliae* ATCC 14706	90.60/*Ochrobactrum soli* BO-7
AAI calculator (%/vs Taxa)	99.11/*Pseudomonas protegens* CHA0	54.11/*Paenibacillus popilliae* ATCC 14706	94.64/*Ochrobactrum soli* BO-7
MiGA - AAI (%/vs Taxa)	98.92/*Pseudomonas protegens* NZ CP022097	64.63/*Paenibacillus pinisoli* GCA_003605435.1	95.0/*Ochrobactrum teleogrylli* GCA_006376685.1
dDDH (d4 in %/Diff %GC/vs Taxa)	89.9/0.04/*Pseudomonas protegens* CHA0	38/13.2/*Paenibacillus popilliae* ATCC 14706	40.8/0.06/*Ochrobactrum soli* BO-7
Species	*Pseudomonas protegens*	*Pristimantibacillus lignocellulolyticus* gen. nov., sp. nov.	*Ochrobactrum gambitense* sp. nov.
GenBank Accession Numbers	CP097898	CP097899	CP098020

*MiGA* The Microbial Genomes Atlas, *TYGS* Type Strain Genome Server, *ANIb*a average nucleotide identity based on BLAST, *ANIm* average nucleotide identity based on MUMmer, *AAI* average amino acid identity, *dDDH* digital DNA:DNA hybridization.

### Novel bacterial taxa found in the MELMC

**Fig. 2:**
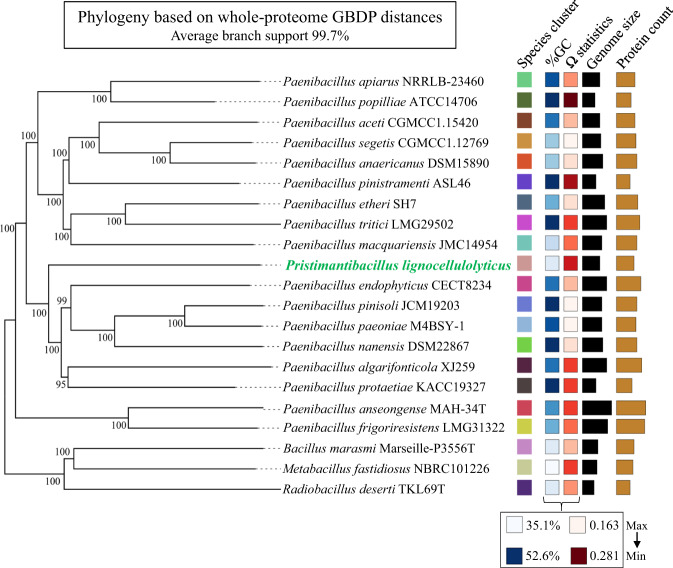
Phylogeny based on whole-proteome data of MAG5. The figure was retrieved from Type Strain Genome Server (TYGS). Tree inferred with FastME v2.1.6.1 from whole-proteome-based GBDP distances. The branch lengths are scaled via GBDP distance formula d5. Branch values are GBDP pseudo-bootstrap support values >60% from 100 replications, with average branch support of 91.9%. The tree was midpoint-rooted.

To determine the taxonomic origin and phylogenomic placement of the three high-quality and circularized MAGs, different approaches and genomic metrics were used. Regarding MAG1, all metrics highly support its affiliation to *Pseudomonas protegens*. On the other hand, MAG4 and MAG5 did not have ANI values (between 68 and 90%) or dDDH percentages (≤41%) indicative of classification in currently named species. The AAI percentages indicated that MAG4 could be closely related to *Ochrobactrum teleogrylli* (95.0%) and MAG5 distantly related to *Paenibacillus pinisoli* (64.6%) (Table 1 and Supplementary Table 2). The taxonomic affiliations of MAG5 and MAG4 to *Paenibacillus* and *Ochrobactrum* genera, respectively, were supported by 16S rRNA phylogeny (Supplementary Fig. S1). In the case of MAG5, a phylogenomic tree based on whole-proteome data and GBDP distances revealed a poorly supported clade corresponding to a species cluster, including the type strains *Pa. endophyticus, Pa. pinisoli, Pa. paeoniae, Pa. nanensis*, and *Pa. algarifonticola* (Fig. 2). However, MAG5 showed lower values of ANIm (87.7% with *Pa. popilliae*) and AAI (64.6% with *Pa. pinisoli*), suggesting that it belongs to a novel genus within the order Caryophanales (“Bacillales”; *p* value: 0.0018, highest taxonomic rank with *p* value ≤0.5 based on MiGA) (Table 1). Following the rules of the SeqCode, this genus is named here *Pristimantibacillus* (Pris.ti.man.ti.ba.cil’lus) (N.L. masc. n. *Pristimantis*, from Pristimantis natural reserve, where soil samples were taken to select the minimal lignocellulolytic bacterial consortium including a member of this taxon; L. masc. n. *bacillus*, little staff, a rod; N.L. masc. n. *Pristimantibacillus*, a rod-shaped cell from Pristimantis natural reserve); a genus established on the basis of MiGA taxonomic novelty analyses, AAI, dDDH, 16S rRNA gene phylogenetic reconstruction, and phylogenomic analyses and is classified as a member of the Paenibacillaceae family (*p* value: 0.221, MiGA). The type species of the genus is *Pristimantibacillus lignocellulolyticus* (lig.no.cel.lu.lo.ly’ti.cus) (N.L. neut. n. *lignocellulosum*, lignocellulose; N.L. masc. adj. *lyticus*, (from Gr. masc. adj. *lytikos*) able to dissolve; N.L. masc. adj. *lignocellulolyticus*, capable of degrading lignocellulose as part of a lignocellulolytic bacterial consortium). The species is established on the same basis as the genus and the type material is the MAG5, deposited in NCBI assembly with accession number CP097899. As for MAG4, MiGA results suggested that it belongs to a novel species within the genus *Ochrobactrum* (*p* value: 0.061), for which the name *Ochrobactrum gambitense* (gam.bi.ten’se) (N.L. neut. adj. *gambitense*, of Gambita, the municipally where soil samples were taken to select the minimal lignocellulolytic bacterial consortium "MELMC) is proposed. The species is established on the basis of MiGA taxonomic novelty analysis, and the type material is the MAG4, deposited in NCBI assembly with accession number CP098020. The names proposed here have been submitted to the SeqCode registry with accession https://seqco.de/r:xwx6hrsf.

### Comparison of 16S rRNA gene sequences from bacterial isolates, MAGs and ASVs

**Fig. 3:**
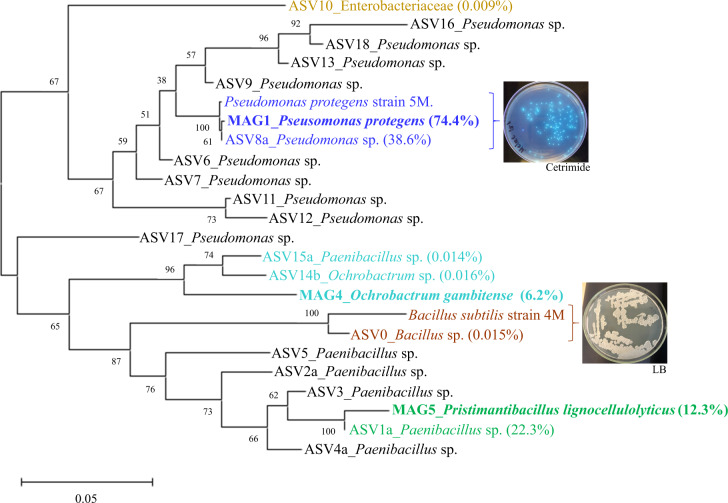
Neighbor-Joining phylogenetic tree based on ribosomal 16S rRNA sequences obtained from metagenome-assembled genomes (MAGs), amplicon-sequence variants (ASVs) reported by Diaz-Garcia et al. [13], and isolated bacterial axenic cultures from the MELMC. A copy of the 16S rRNA gene was randomly selected from each MAG to build the phylogenetic tree. Bootstrap tests (1000 replicates) are shown next to the branches and evolutionary distances are in units of the number of base differences per site. At right, we display the colonies of the recovered bacterial axenic cultures (*Pseudomonas protegens* and *Bacillus subtilis*).

A total of 19 bacterial pure cultures, with different colony morphologies, were recovered from the MELMC. From those, 17 isolates were taxonomically classified using their 16S rRNA gene sequences. Only two different species were identified (twelve isolates belonged to *Ps. protegens* and five to *Bacillus subtilis*) (Supplementary Table 3). Previously, 16S rRNA amplicon sequencing analysis revealed that the MELMC was mostly composed of two species (*Pseudomonas* sp. and *Paenibacillus* sp.), with additional ASVs found in very low abundance (<0.01%) [13]. To determine if the MAGs match one of the recovered axenic cultures, and to compare them with the reported ASVs, a phylogenetic tree was constructed using all the 16S rRNA gene sequences (Fig. 3). The results indicated that MAG1 was recovered as a pure culture (type strain 5 M). Based on total metagenomic mapped reads, the relative abundance of MAG1 was 74% compared to 38.6% obtained in the 16S rRNA amplicon sequencing analysis. The complete 16S rRNA gene sequence from MAG5 was highly similar to ASV1a (with a relative abundance of 22.3%). The MAG5 was the second most abundant MELMC member (12.3%). Moreover, MAG4, with a relative abundance of 6.2%, showed high similarity with two reported ASVs found at very low abundance (<0.016%). MAG4 and MAG5 were not associated with any bacterial isolate. However, an axenic culture of *B. subtilis* was recovered, and it showed similarity with an ASV0 found at very low abundance (0.015%) within the MELMC (Fig. 3).

### Identification of CAZymes involved in lignocellulose degradation

**Fig. 4:**
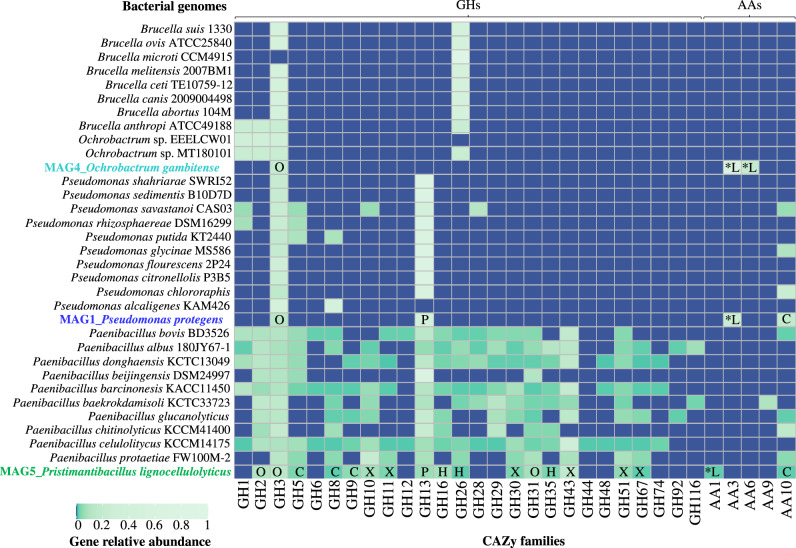
Relative abundance of CAZy families involved in plant polymer transformation found in the retrieved MAGs from the MELMC. Comparison was performed using the CAZyme profiles of 30 close-bacterial genomes. Heatmaps were constructed using normalized values (by number of hits per genome). The labels within squares represent the type of polymers where enzymes could act: O oligosaccharides, C cellulose, L lignin, X xylan, P pectin. Asterisks represent CAZy families uniquely found in the MAGs from the MELMC.

The CAZyme profile associated with deconstruction of lignocellulose [8, 17] was analyzed for the three MAGs obtained from the MELMC, and compared with 30 other closely related bacterial genomes (Fig. 4). The results showed that *Paenibacillus* species contains the highest potential to deconstruct plant polysaccharides due to the high abundance and diversity of many glycoside hydrolases (GHs) families. In particular, MAG5 contained a higher number of genes to deconstruct cellulose (e.g., families GH5, GH8, GH9, and AA10) and xylan (e.g., families GH10, GH11, GH30, GH43, GH51, and GH67) compared to MAG1 and MAG4. Clearly, *Pseudomonas* and *Ochrobactrum* species had a smaller potential to deconstruct plant polysaccharides. Within the *Pseudomonas* species, there was a common potential for the degradation of oligosaccharides and pectin, which is reflected by the presence of families GH3 and GH13. Interestingly, MAG1 and MAG4 contain genes from families AA3 (involved in the oxidation of alcohols or carbohydrates) and AA6 (involved in the catabolism of aromatic compounds). In addition, MAG5 contained genes of the AA1 family (i.e., laccases) (Fig. 4). These former AA families could be involved in lignin transformations.

### Lignin catabolism profile of the MELMC

We explored the presence and abundance of 92 types of proteins involved in the transformation of lignin [51] within the three MAGs. In total 66, 21, and 22 genes encoding 40 types of lignin-transforming enzymes were found within MAG1, MAG5, and MAG4, respectively (Supplementary Table 4). Interestingly, MAG1 (*Ps. protegens*) shows the largest potential to depolymerize lignin, reflected by the presence of genes encoding glutathione S-transferases (EC 2.5.1.18), catalases (EC 1.11.1.6), and glutathione peroxidases (EC 1.11.1.9). These enzymes could also be expressed in response to oxidative stress. In particular, MAG1 encodes genes involved in the catabolism of lignin monomers through the beta-ketoadipate (e.g., *pca* genes) and protocatechuate cleavage pathways (e.g., *lig* and *mhp* genes). In addition, genes involved in catechol (*catABC*) and vanillin (*vanAB* genes) catabolism were also found in MAG1 (Supplementary Table 4). Based on KEGG database and RAST annotation, other regulators (PerR and CtsR) and genes (e.g., the *mhqA* that encodes a hydroquinone-specific dioxygenase) involved in thiol-specific oxidative stress response and associated with the consumption of aromatic compounds, such as 2-methylhydroquinone or catechol, were found in *Pr. lignocellulolyticus* (MAG5).

### Potential of the MELMC to transform plastics and its derived compounds

**Fig. 5:**
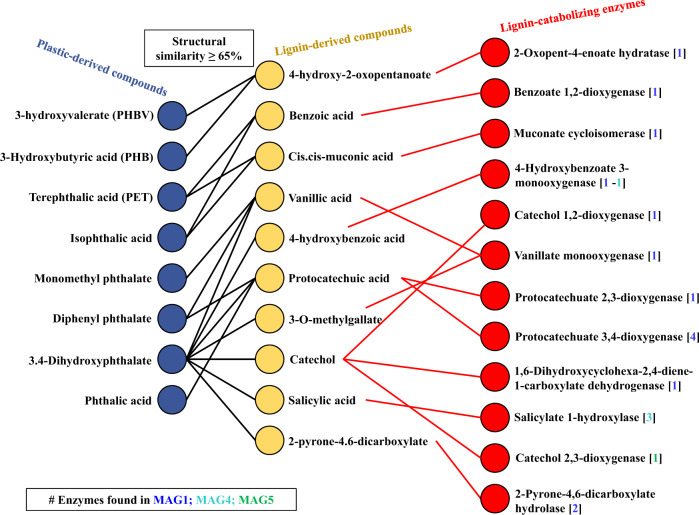
Tripartite network analysis. Colored circles indicate from left to right: plastic-derived chemical compounds (PDCC) (blue), lignin-derived chemical compounds (LDCC) (yellow), and lignin-catabolizing enzymes (red). Black edges represent SMILES similarity values (based on Tanimoto coefficients) greater than 65%. Red edges connect lignin-derived substrates with enzymes that use them as substrate. The number of genes found in each MAG is indicated in squared brackets.

Based on the functional annotation using PMBD and PlasticDB databases, it was observed that MAG1 contains genes associated with the transformation of polyurethane (PU), polybutylene adipate terephthalate (PBAT), and low-density polyethylene (LDPE) (Table 2). MAG4 showed a genomic capacity to produce phthalate dioxygenases. In addition, MAG1 and MAG4 have the genomic potential to produce 3HV-dioxygenases, probably involved in the intracellular metabolism of poly(3-hydroxybutyrate-co-3-hydroxyvalerate) (PHBV). Based on the structural features (using SMILES) between LDCC and PDCC, a method to quantify similarity using Tanimoto coefficients was developed. Here, 43 PDCC were pairwise compared with each of the 70 LDCC (Supplementary Table 5). From this analysis, we could predict which lignin-transforming enzymes could be active on PDCC. The tripartite network (Fig. 5) displayed three main cases: (i) PDCC that are similar to one LDCC, which in turn is catabolized by a single enzyme, (e.g, monomethyl phthalate); (ii) a PDCC similar to two or more LDCC, which are substrates for different enzymes (e.g., 3,4-dihydroxyphtalate); (iii) two PDCC that are similar to one LDCC, catabolized by a single enzyme (e.g., 3-hydroxyvalerate and 3-hydroxybutyric acid). Finally, the tripartite network showed that *Ps. protegens* (MAG1) is expected to catabolize monomers from PHBs (e.g., PHB and PHBV) and PET (e.g., terephthalic acid), and other chemical compounds commonly used as plasticizers (e.g., phthalates). Finally, MAG4 encodes two enzymes that could transform 3,4 hydroxyphthalate (4-hydroxybenzoate 3-monooxygenase and salicylate 1-hydroxylase), an intermediate compound in phthalates degradation (Fig. 5).

**Table 2:** Functional annotation of the MAGs using PMBD and PlasticDB databases.

		Number of hits		
Enzyme type	Database (annotation)	MAG_1	MAG_4	Plastic to be catalyzed
Polyurethanase	PMBD (BLASTX)	14	0	PU
Polyurethanase A	PMBD (BLASTX)	8	0	PU
Polyurethanase B	PMBD (BLASTX)	2	0	PU
Polyurethanase esterase A	PMBD (BLASTX)	1	0	PU
Phthalate dioxygenase reductase	PMBD (BLASTX)	0	1	Phthalate
		Number of proteins		
Polyurethanase	PlasticDB (BLASTP)	2	0	PU
Polyesterase	PlasticDB (BLASTP)	1	0	PBAT
3HV_dehydrogenase	PlasticDB (BLASTP)	1	1	P3HV-Oligomers/PHBV-Oligomers
Alkane monooxygenase	PlasticDB (BLASTP)	1	0	LDPE

*PU* polyurethane, *PBAT* polybutylene adipate terephthalate, *LDPE* low-density polyethylene, *P3HV* poly(3-hidroxivalerato), *PHBV* poly(3-hydroxybutyrate-co-3-hydroxyvalerate).

### Catabolism and production of polyhydroxyalkanoates in *Ps. protegens* (MAG1)

**Fig. 6:**
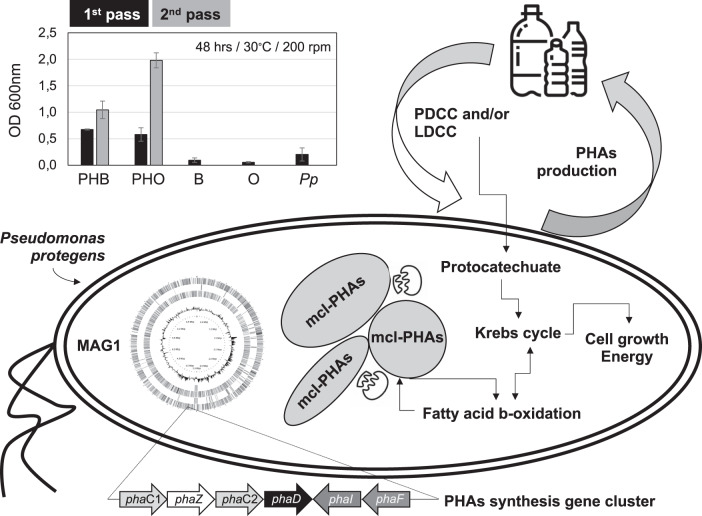
Schematic figure to represent the PHAs metabolism in *Ps. protegens* (MAG1) and its potential for plastic upcycling. In the left-up, we display the growth (assessed by OD_600nm_) values obtained on PHB and PHO as the sole carbon source; B and O are controls without bacterial inoculum and *Pp* is the controls without PHAs. Cluster of genes (arrows depending on their transcription direction) associated with PHA synthesis (and found in MAG1) are at the figure bottom. In right-middle, we display how plastic monomers or plasticizers can be metabolized thought protocatechuate pathways allowing the growth of *Ps. protegens* and synthesis of PHAs. These PHAs can be catabolized and synthetized through fatty acid beta-oxidation pathway.

It was observed that *Ps. protegens* (i.e., strain M5; MAG1) can grow on PHB and PHO as sole carbon sources, reaching an average OD_600nm_ of 0.7 (PHB) and 0.6 (PHO) after 48 h of incubation in the first pass. Upon transfer to fresh media containing nanoparticles (2nd transfer), *Ps. protegens* grew faster, showing OD_600nm_ average values of 1.0 and 2.0 for PHB and PHO, respectively, compared to the first batch at the same incubation time (Fig. 6). When looking into its genomic potential, MAG1 encode some extracellular lipases predicted to depolymerize PHAs (Supplementary Table 6). Furthermore, several genes associated with PHA catabolism were found in MAG1, including an intracellular PHO depolymerase (encoded by *pha*Z) (Fig. 6), and others encoding enzymes involved in fatty acids (PHA-related substrates) metabolism through β-oxidation pathway. For instance, acyl-CoA synthetase (EC 6.2.1.3), acyl-CoA dehydrogenase (EC 1.3.1.8), enoyl-CoA hydratase (EC 4.2.1.17), 3-hydroxybutyryl-CoA dehydrogenase (EC 1.1.1.157), and 3-ketoacyl-CoA thiolase (EC 2.3.1.16). Moreover, a cluster of genes (*phaC1ZC2D*), well conserved among the medium-chain length-PHA (mcl-PHA) producer strains like *Ps. putida* KT2440, was found within MAG1. The cluster is composed of *phaC1* and *phaC2* genes encoding two class II synthases, *phaD* encoding a TetR-like transcriptional regulator; and *phaI* and *phaF* encoding structural proteins known as phasins that are highly relevant in the production and regulation of PHAs (Fig. 6).

## Discussion

The selection and design of plant biomass-degrading microbial consortia (PBDC) has been traditionally carried out to reach three main goals: (i) to reduce the complexity of wild-type communities, aiming for a better understanding of the ecology, enzymology, and dynamics behind lignocellulose transformation [23]; (ii) to design a microbial platform for the production of enzyme cocktails useful in biorefineries [17]; and (iii) to produce commodity chemicals, biopolymers, biofuels or pharmaceutical compounds in a consolidated bioprocess [66]. For the first goal, minimal and effective PBDC have been designed using the “bottom-up” strategy [67]. For instance, a tripartite fungal-bacterial consortium has been exhaustively explored in different studies [15, 68, 69]. In contrast, information and knowledge on the selection of minimal PBDC using the “top-down” approach is limited. In this regard, we have developed an innovative enrichment method to select a MELMC from Colombian Andean Forest soils [13].

Metagenomic surveys have allowed us to extract and analyze meaningful information about lignocellulolytic microbial communities from different natural environments [70, 71]. Recently, genome-resolved metagenomics has been used to characterize different polymer-degrading consortia [20, 72]. In this regard, Peng et al. [73] found 719 high-quality MAGs (unique at the species level) from PBDC retrieved from goat guts. In our study, the first relevant observation was that the MELMC is composed mostly of three species (Fig. 1), instead of two as 16S rRNA amplicon sequencing showed [13]. The relative abundance of the three high-quality MAGs (MAG1, MAG4, and MAG5) obtained from the MELMC accounts for more than 90% (Fig. 2). Two of them belonged to not-yet cultured (as axenic cultures) novel bacterial taxa (Table 1 and Fig. 2). Third generation sequencing technologies (e.g., PacBio-HiFi and Nanopore) and improved bioinformatics tools have allowed us to discover several novel microbial species, represented by MAGs, in different natural environments [74]. Unfortunately, many of these MAGs come from uncultivable microorganisms which cannot be systematically named following the rules of the International Code of Nomenclature of Prokaryotes [44]. Therefore, a roadmap for naming uncultivated microorganism has been proposed, in which genome sequences could serve as the type material for naming prokaryotic taxa [75]. Based on various genomic metrics (such as ANI and AAI), MAG5 represent a novel bacterial genus within the Paenibacillaceae family, for which the name *Pristimantibacillus lignocellulolyticum* is proposed here, following the rules of the SeqCode. In this case, the suffix *bacillus* is used to describe the morphology of the cells attached to lignocellulosic biomass, as found previously by scanning electron microscopy [13]. Moreover, MAG4 was classified as a novel species within the genus *Ochrobactrum*, which is a soil-dwelling bacterium [76]. Notably, the genus *Ochrobactrum* is possibly a later heterotypic synonym of the pathogen genus *Brucella* [47], but we maintain the designation for consistency with the named species closest to MAG4, and in accordance with Moreno et al. [77].

Interestingly, prophage sequences were found in the three high-quality MAGs retrieved from the MELMC. Among them, a complete viral sequence associated with the *Salmonella* phage 118970_sal3 was detected within MAG1 (Supplementary Table 1). We hypothesize that this phage could negatively impact the enterobacterial populations during the MELMC development, decreasing their abundance as was observed by Díaz-Garcia et al. [13]. However, the ecological relevance and the functional roles of bacteriophages within lignocellulolytic microbial communities are still unclear. Thus, further studies must explore the viral communities in these PBDC, as is reported for soils [78]. Moreover, the most abundant member of the MELMC (*Ps. protegens;* MAG1) was isolated as axenic culture. However, the other two members were not recovered as pure isolates by using commercial agar media (i.e., LB, R2A, and Cetrimide). Both *O. gambitense* and *Pr. lignocellulolyticus* could grow and maintain in a tripartite consortium, probably due to their metabolic interdependences between the MELMC species [79]. This MELMC could be an excellent system for further studies of culturomics, as is described for other microbial communities [80]. Nutritional requirements to design a suitable culture medium for isolation of *O. gambitensis* and *Pr. lignocellulolyticum* can be identified using the MAG4 and MAG5 sequence information. Moreover, a very low abundance (0.015%) MELMC member (*B. subtilis*) was isolated in an axenic culture (Fig. 2). We hypothesize that LB agar and incubation conditions favored the growth of this bacterium. Here, we suggested that *B. subtilis* could be present as a remnant species after the dilution-to-extinction process [13], being metabolically inactive within the MELMC, but it could be an excellent partner of *Ps. protegens* for the transformation of fossil-based plastics, as is reported by Roberts et al. [32].

Regarding lignocellulose transformation, *Pr. lignocellulolyticus* contains the biggest genomic capacity to deconstruct plant polysaccharides, in particular cellulose and xylan (Fig. 3), similar to what has been reported for *Paenibacillus* species [81]. On the other hand, *Ps. protegens* has the largest genomic potential to depolymerize lignin and catabolize its derived aromatic compounds (Supplementary Table 4) [82]. However, *Pr. lignocellulolyticus* could aid in the depolymerization process by producing laccases (from the AA1 family). Based on this evidence, we posit that both enzymatic synergism and division of labor are common features within the MELMC, similar to other PBDC [51, 68]. Following cheminformatic principles, which have been used in pharmacology and drug discovery [83], quantitative structural relationships (using SMILES and Tanimoto coefficients) between LDCC and PDCC were obtained (Fig. 5 and Supplementary Table 5). From these analyses, combined with target-specific functional annotation of MAGs, we propose that *Ps. protegens* could transform PBAT (a biodegradable fossil-based plastic). A predicted polyesterase [84] encoded in MAG1 could depolymerize PBAT to its units: adipic acid, 1,4-butanediol, and terephthalic acid; and then terephthalic acid could be incorporated into *Ps. protegens* metabolism. Based on the tripartite network (Fig. 5), cis-, cis-muconic acid, and benzoic acid were structurally similar to terephthalic acid. It is reported that cis-, cis-muconic acid can be chemically converted to terephthalic acid for the production of bio-PET from lignin [85]. Therefore, it could be anticipated that the catabolism of terephthalic acid (one of the building blocks of PET and PBAT) could be accomplished by the action of the muconate cycloisomerase (EC 5.5.1.1) or benzoate 1,2-dioxygenase (EC 1.14.12.10), which have catalytic activity on cis-, cis-muconic acid and benzoic acid, respectively [59, 86]. In fact, muconate cycloisomerase has been involved in the catabolism of aromatic intermediates obtained within the transformation of plastic additives [87]. Moreover, in *Ps. putida* KT2440 the gene *ben*A encoding a benzoate 1,2-dioxygenase share a similarity of 35% with the gene *tph*A2 encoding a terephthalate 1,2-dioxygenase in *Ps. umsogensis* GO16 [88]. Uptake and catabolism of terephthalic acid has been described in other organisms, including different species of *Pseudomonas*, and proceeds via protocatechuate, a common intermedia in aerobic aromatic catabolism [89]. Notably, *Ps. protegens* (MAG1) contains the gene encoding a vanillate monooxygenase (EC 1.14.13.82), an enzyme involved in the catabolism of vanillate into protocatechuate [59]. This enzyme could have activity on monomethyl phthalate (a common plasticizer agent), who shares a structural similarity of nearly 70% with vanillate (Supplementary Table 5). To corroborate the structural similarities between LDCC and PDCC, additional molecular fingerprints would be helpful [90]. However, growth experiments can determine if *Ps. protegens* can catabolize some PDCC, and isotope-labeling experiments [91] could be also helpful to determine the depolymerization of PBAT and PU by this bacterium.

PHAs are versatile bio-based and biodegradable polymers that can be useful to replace fossil-based plastics [30]. As a significant finding in our study, we found that *P. protegens* (MAG1) harbors the *pha* cluster for the synthesis and accumulation of mcl-PHAs, like PHO (Fig. 6), a capability widespread in *Pseudomonas* species. Intracellular PHA can be hydrolyzed by the action of the PhaZ proteins, maintaining the turnover inside of the cells through synthesis and depolymerization cycles [92, 93]. In *Ps. protegens* a *pha*Z gene was found within the *pha* cluster. Moreover, PHA is released into the extracellular environment after the death of PHA-producing microorganisms. This polymer can be deconstructed into its units of 3-hydroxyalkanoic by the action of extracellular PHA depolymerases (e-PHA). Most of the e-PHA have specificity for PHB (short-chain length). However, some e-PHA, isolated from *Bdellovibrio*, *Pseudomonas*, or *Streptomyces* species, can act on mcl-PHA, such as PHO [94, 95]. In this study, we incubated the axenic culture (strain M5) of *Ps. protegens* with PHB or PHO nanoparticles as the sole carbon source, observing growth on both substrates. Despite the apparent absence of e-PHA within MAG1, it has been described that *Pseudomonas* species can degrade PHA using extracellular lipases, like those encoded in MAG1 (Supplementary Table 6). These findings suggest that *P. protegens* contains the genomic potential to synthesize mcl-PHAs and produces enzymes to depolymerize PHB and PHO; catabolizing its oligomers or monomers for growth (Fig. 6). Whether these oligo- and monomers could be re-polymerized into PHA, as suggested by some studies, still needs further and systematic investigation [96]. From a biotechnological perspective, PHA depolymerases are interesting due to their capacity to produce enantiopure R-hydroxycarboxylic with potential applications for the synthesis of antibiotics, and for conjugation of anti-cancer compounds [97].

In conclusion, the combined “top-down” enrichment strategy is an excellent approach to select a minimal and effective polymer-transforming microbial consortium. Here, the PacBio-HiFI metagenome sequencing was useful to reconstruct the genomes of the three most abundant MELMC species, unveiling two novel bacterial taxa involved in lignocellulose transformation. One of them (*Pr. lignocellulolyticus*) contains a wide genomic potential to deconstruct plant polysaccharides. Thus, this species can be a source of enzymes useful to improve the saccharification process in biorefineries. Moreover, the MELMC is dominated by *Ps. protegens*, which was able to isolate in axenic culture, displaying the highest genomic potential to (i) catabolize LDCC; (ii) depolymerize plastics, such as PBAT, PE, and PU; (iii) catabolize terephthalate and other PDCC, such as phthalates; and (iv) produce mcl-PHAs and degrade extracellular PHAs. As a key perspective, further studies can focus on the production of biodegradable plastics (i.e., PHAs), by *Ps. protegens*, using PDCC (e.g., terephthalic acid) and LDCC (Fig. 6), as it has been proposed for *Ps. umsongensis* and *Ps. protegens* strains [88, 98, 99].

## Supplementary information

Supplementary Figure and Table Legends

Figure S1

Table S1

Table S2

Table S3

Table S4

Table S5

Table S6

## Data Availability

All data (raw and assemblies) are connected through the same BioProject ID, namely PRJNA842362.
