# Supplementary material for: Novel bacterial taxa in a minimal lignocellulolytic consortium and their potential for lignin and plastics transformation
Source: ISME Commun. 2022 Sep 26;2:89. doi: 10.1038/s43705-022-00176-7 (PMC9723784; doi:10.1038/s43705-022-00176-7)
Supplement: Supplementary file 1 — Supplementary Figure and Table Legends [file 43705_2022_176_MOESM1_ESM.docx]

**SUPPLEMENTARY FIGURES LEGENDS**

**Fig S1.** Phylogenetic tree based on 16S rRNA gene sequences obtained from A) MAG5 and B) MAG4, and its relationship with related species. ML tree inferred under the GTR+GAMMA model and rooted by midpoint-rooting. The branches are scaled in terms of the expected number of substitutions per site. The numbers above the branches are support values when larger than 60% from ML (left) and MP (right) bootstrapping.

**SUPPLEMENTARY TABLES**

**Supplementary Table S1.** Prophage sequences retrieved from the MAGs

**Supplementary Table S2.** AAI values found in the MAGs using the MiGA webserver

**Supplementary Table S3.** BLASTn results of the bacterial 16S rRNA sequences obtained from axenic cultures

**Supplementary Table S4.** Genes (KO identifiers) encoding enzymes and their probable function or metabolic pathway within the lignin transformation processes for each MELMC member.

**Supplementary Table S5. Tab S1)** Chemical compounds associated with lignin and synthetic plastic polymers in the Simplified Molecular Input Line Entry Specification (SMILES) format. **Tab S2)** Similarity matrix between lignin and plastic-derived compounds (i.e., LDCC and PDCC). **Tab S3)** Enzymes involved in lignin transformation and its lignin-derived substrates.

**Supplementary Table S6.** Peptide signals associated with lipases found in the KEGG annotation for the MAGs from the MELMC.
