# Supplementary figures and images for: Novel bacterial taxa in a minimal lignocellulolytic consortium and their potential for lignin and plastics transformation
Source: ISME Commun. 2022 Sep 26;2:89. doi: 10.1038/s43705-022-00176-7 (PMC9723784; doi:10.1038/s43705-022-00176-7)

## Slide 1
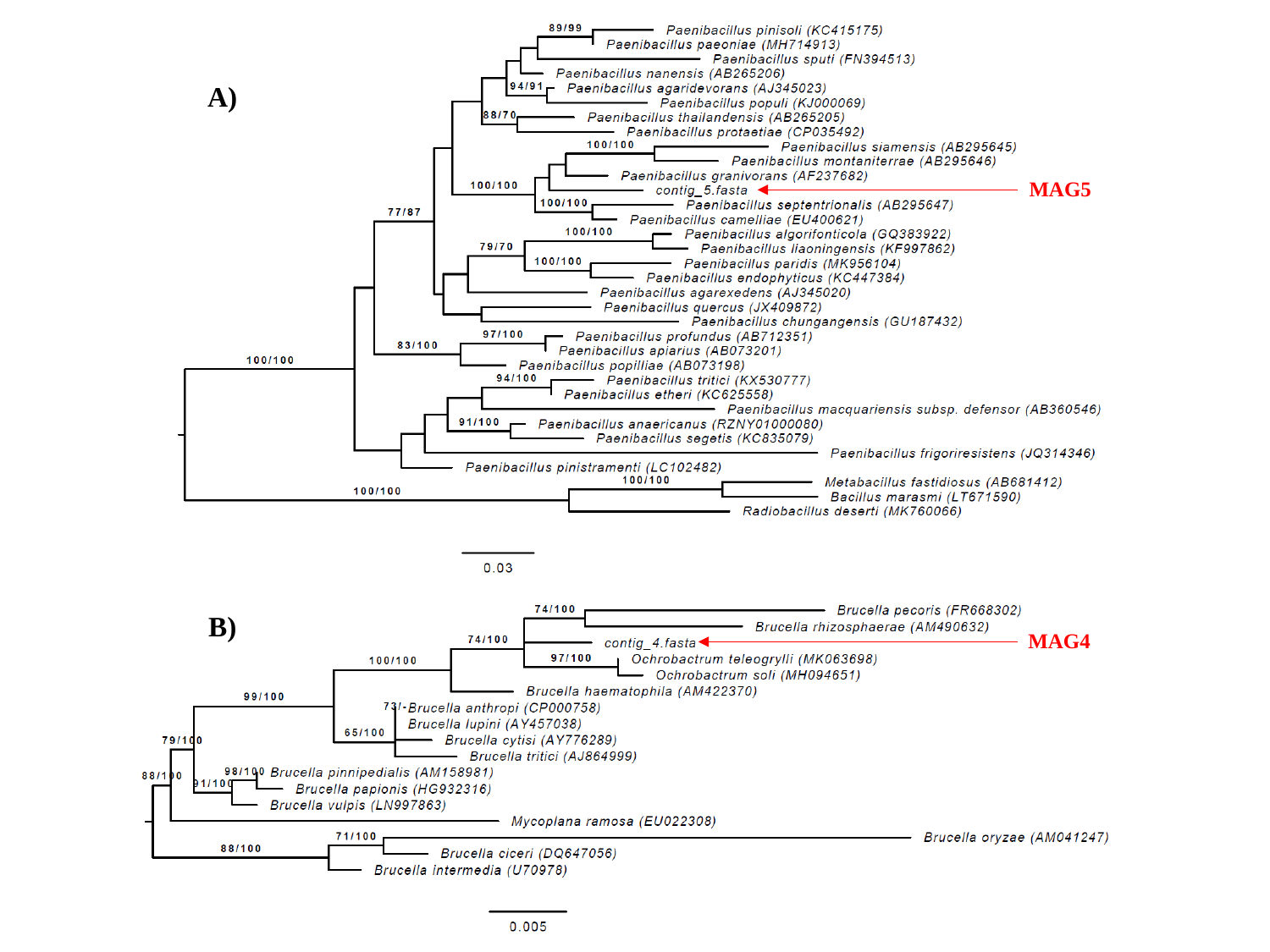

A)
MAG5
B)
MAG4

Supplement: Supplementary file 2 — Figure S1 [file 43705_2022_176_MOESM2_ESM.pptx]
